# Resolvin D1 Modulates the Inflammatory Processes of Human Periodontal Ligament Cells via NF-κB and MAPK Signaling Pathways

**DOI:** 10.3390/biomedicines13123038

**Published:** 2025-12-10

**Authors:** Jing Yan, Jiazheng Cai, Xiaojing Pan, Si Li, Christopher Graham Fenton, Kristin Andreassen Fenton, Alpdogan Kantarci, Yaxin Xue, Ying Xue, Zhe Xing

**Affiliations:** 1 School/Hospital of Stomatology, Lanzhou University, Lanzhou 730000, China; 2 Department of Medical Biology, Faculty of Health Sciences, UiT the Arctic University of Norway, 9037 Tromsø, Norway; kristin.fenton@uit.no; 3 Genomics Support Centre Tromsø (GSCT), Department of Clinical Medicine, Faculty of Health Sciences, UiT the Arctic University of Norway, 9037 Tromsø, Norway; 4 Department of Developmental and Surgical Sciences, School of Dentistry, University of Minnesota, Minneapolis, MN 55455, USA; 5 School of Dental Medicine, Harvard University, Boston, MA 02115, USA; 6 Computational Biology Unit, Department of Informatics, University of Bergen, 5008 Bergen, Norway; 7 Department of Clinical Dentistry, Faculty of Health Sciences, UiT the Arctic University of Norway, 9037 Tromsø, Norway; 8 Key Laboratory of Dental Maxillofacial Reconstruction and Biological Intelligence Manufacturing, Lanzhou University, Lanzhou 730000, China

**Keywords:** periodontal ligament cells, Resolvin D1, RNA-seq, NF-κB, MAPK

## Abstract

**Objectives:** Periodontitis is a multifactorial inflammatory disease initiated by pathogenic bacteria, such as *Porphyromonas gingivalis*. Resolvin D1 (RvD1) plays a pivotal role in inflammation resolution. This study aimed to identify the mechanism of the regulatory effects of RvD1 on the inflammatory response of human periodontal ligament cells (hPDLCs). **Methods:** To investigate the mechanism of RvD1’s impact on the hPDLCs, RNA-sequencing (RNA-seq) was used and differentially expressed genes (DEGs) were identified. Gene Ontology (GO) and Kyoto Encyclopedia of Genes and Genomes (KEGG) analyses were performed to assess the signaling pathways in which NF-κB and MAPK were determined to play a significant role. Alterations in NF-κB and MAPK pathways were verified by immunofluorescence (IF), quantitative real-time PCR (qRT-PCR), and Western blotting (WB). The expression of RvD1 and lipoxin A4/formyl peptide receptor 2 (ALX/FPR2) was assessed by IF and WB. Inflammatory cytokine interleukin (IL) 6 and IL-1β release was measured by ELISA. **Results:** GO and KEGG analyses indicated that RvD1 regulates the inflammatory process in PDLCs primarily via TLR4-MyD88-mediated NF-κB and MAPK signaling. RvD1 suppressed lipopolysaccharide (LPS)-induced TLR4 and MyD88 expression, inhibited phosphorylation of NF-κB p65 and its inhibitor IKBKB, and attenuated phosphorylation of p38 MAPK, ERK, and JNK. ALX/FPR2 was expressed on hPDLCs and was further upregulated upon treatment with RvD1. RvD1 significantly down-regulated the IL-6 and IL-1β levels in LPS-stimulated hPDLCs. **Conclusions:** RvD1 regulates the inflammatory response of LPS-stimulated hPDLCs by the TLR4-MyD88-MAPK and TLR4-MyD88-NF-κB signaling pathways, suggesting the potential role of RvD1 in restoring periodontal tissue homeostasis by regulating PDLC response to inflammatory and infectious stimuli.

## 1. Introduction

Periodontitis is a chronic inflammatory disease initiated by microbial biofilms, in which Gram-negative anaerobic bacteria such as *Porphyromonas gingivalis* play a key pathogenic role [1]. In response to these biofilms, periodontal tissue induces an acute inflammatory reaction. The resolution of acute inflammation is mediated by cytokines and specialized proresolving lipid mediators (SPMs). Notably, SPMs facilitate the resolution phase of inflammation and enhance the regenerative potential of human periodontal ligament cells (hPDLCs), thereby restoring homeostasis [2,3,4]. However, when excessive acute inflammation fails to resolve or microbial proliferation continues, the inflammation transitions into a chronic state, resulting in disrupted periodontal homeostasis and alveolar bone resorption [5,6,7].

Lipopolysaccharide (LPS), a major structural component of the outer membrane of *P. gingivalis*, is recognized by Toll-like receptor 4 (TLR4), thereby triggering inflammatory signaling cascades [8,9]. TLR4 recruits adaptor proteins such as TIRAP and MyD88, which subsequently activate the downstream NF-κB and MAPK signaling pathways, culminating in the release of chemokines and pro-inflammatory cytokines [10,11,12,13].

SPMs, including lipoxins, resolvins, protectins and maresins, play key roles in restoring tissue homeostasis by restraining leukocyte infiltration and suppressing pro-inflammatory signals [14,15]. Resolvin D1 (RvD1), a member of the D-series resolvins, is enzymatically derived from docosahexaenoic acid (DHA) through a 17-hydroperoxy intermediate catalyzed by 15-lipoxygenase [16]. Increasing evidence indicates that RvD1 possesses potent anti-inflammatory and antioxidant properties, demonstrating therapeutic potential in various inflammatory conditions such as acute lung injury, diabetic retinopathy, and periodontitis [17,18,19]. Furthermore, a previous in vivo study established that the RvD1-GelMA complex alleviates inflammation and promotes tissue regeneration in a rat model of periodontitis [20]. RvD1 primarily exerts its biological effects through ALX/FPR2, which can be activated by both pro-inflammatory ligands (such as fMLP) and pro-resolving ligands (such as RvD1 and LXA4), thereby mediating distinct cellular responses [4,21,22]. Studies have shown that ALX/FPR2 on periodontal ligament cells can respond to resolving mediators upon activation by RvD1 [21]. Furthermore, RvD1 inhibits key pro-inflammatory pathways through ALX/FPR2. The downstream signaling network mediated by ALX/FPR2 includes the NF-κB pathway, the MAPK pathway, and the IRAK1/TRAF6 pathway [21,23,24].

Studies have demonstrated that RvD1 promotes wound healing and the proliferation of periodontal fibroblasts while suppressing the production of pro-inflammatory cytokines [4,25,26]. However, its comprehensive transcriptional regulatory mechanisms in hPDLCs remain unclear. The effects of RvD1 on the relevant signaling pathways of hPDLCs have not yet been fully clarified. Therefore, this study aimed to test the hypothesis that RvD1 regulates the inflammatory and infection-induced responses of hPDLCs to restore the homeostasis of periodontal tissues, and to identify the differentially expressed genes (DEGs) and signaling pathways involved in this regulatory process.

## 2. Materials and Methods

### 2.1. Culture and Characterization of hPDLCs

The isolation of human periodontal ligament cells (hPDLCs) was performed following our previous experimental protocols [25]. Periodontal ligament tissues were collected from six healthy individuals (ages 12–23 years) without any symptoms of periodontal disease [27].

Briefly, periodontal tissues were minced and cultured in a 25 cm^2^ cell culturing flask (Beaver, Guangzhou, China). Outgrowing cells were detached using 0.025% trypsin and maintained at 37 °C in a humidified atmosphere containing 5% CO_2_. The morphology of hPDLCs was examined utilizing a microscope (OLYMPUS, CKX53, Tokyo, Japan), and the cells displayed a characteristic spindle shape. All experiments were conducted using 3–5 passages of hPDLCs, which were cultured in a cell culturing flask at 3 × 10^5^ cells per flask.

For immunocytochemical identification, hPDLCs were seeded in 6-well plates (7 × 10^4^ cells per well). After three PBS washes, the cells were fixed for a duration of 30 min. The primary antibodies included rabbit anti-human vimentin antibody (1:200, Proteintech, Rosemont, IL, USA) and mouse anti-human keratin antibody (ZM-0069, ZSGB-BIO, Beijing, China). And then the cells were incubated with fluorescent secondary antibodies for 1 h. DAPI was used for cell nuclei staining. Finally, an inverted microscope was used to assess the staining and take photographs (OLYMPUS, CKX53, Tokyo, Japan).

### 2.2. Experimental Design

To evaluate the effects of Lipopolysaccharide (LPS) and Resolvin D1 (RvD1) on hPDLCs, the experiments were designed as follows: (a) control group; (b) LPS group; (c) LPS+RvD1 group; (d) RvD1 group. For the LPS group, cells were exposed to LPS (0.1 μg/mL, catalog # L8880; Solarbio^®^, Beijing, China) for 24 h. For the LPS+RvD1 group, LPS (0.1 μg/mL) was added to the flask for 12 h, and then RvD1 (0.1 μg/mL, Cayman Chemical, Ann Arbor, Michigan) was applied for 12 h. For the RvD1 group, RvD1 (0.1 μg/mL) was applied for 12 h. The concentrations of LPS and RvD1 were determined in our previous work [25,28,29].

### 2.3. RNA-Seq and Sequencing Data Analysis

Total RNA was extracted from hPDLCs using TRIzol (AmbionÒ, Austin, TX, USA). RNA concentration was ≥0.1 µg/µL with a total amount > 2 µg per sample. For each group, equal amounts of RNA from three biological replicates were pooled to construct one RNA-seq library. Agilent 2100 Bioanalyzer (Agilent Technologies, Santa Clara, CA, USA) and NanoDrop 2000 (Thermo Fisher Scientific, Waltham, MA, USA) were used to detect the total RNA for each group.

The RNA-seq libraries were sequenced on the Illumina HiSeq platform using a 2 × 150 bp paired-end mode, generating raw reads in FastQ format. Differential gene expression analysis was performed using DESeq2 (v1.10.1), with a fold change of ≥2 and a false discovery rate *p* ≤ 0.05 set as the criteria for statistical significance [30].

Gene Ontology (GO) and Kyoto Encyclopedia of Genes and Genomes (KEGG) pathway enrichment analyses were carried out using the clusterProfiler R package (v2.4.2), applying Fisher’s exact test.

### 2.4. qRT-PCR

Real-time qRT-PCR was performed with UltraSYBR Mixture as described by the manufacturer (Cwbio, Beijing, China). Data were analyzed by the comparative (2^−ΔΔCT^) method. *GAPDH* served as a model gene. In Table S1, the primer sequences are displayed.

### 2.5. WB Analysis

After extracting total protein with RIPA lysis buffer, it was separated by 10% SDS-PAGE and then transferred to a PVDF membrane (PVDF, Amersham^TM^, Freiburg, Germany). The blocked membrane was incubated with the primary antibody overnight. The primary antibodies included: β-actin (1:1000, Proteintech, Rosemont, IL, USA), ALX/FPR2 (1:1000, Abcam, Cambridge, UK), TLR4, MyD88, p65, p-p65, IKBKB, JNK (1:1000, Proteintech, Rosemont, IL, USA), ERK (1:1000, Immunoway, Plano, TX, USA), p-ERK (1:1000, Bioworld, Louis Park, MN, USA), p38 (1:1000, Proteintech, Rosemont, IL, USA), p-p38 (1:1000, Abcam, Cambridge, UK). After washing in PBST 3× 5 min, the membranes were subjected to an incubation for two hours with secondary goat anti-rabbit or goat anti-mouse (1:3000, Proteintech, Rosemont, IL, USA) antibodies. NcmECL Ultra (NCM Biotech, Suzhou, China) was used to visualize protein bands using a chemiluminescence device (VILBER, Marne-la-vallée, France). The imaging data were analyzed using ImageJ software (version 1.46r, Bethesda^®^, Montgomery, MD, USA).

### 2.6. Immunofluorescence (IF) Assays

HPDLCs were cultured in 24-well plates, with 3000 cells per well. After washing three times with cold PBS, the cells were fixed in 4% paraformaldehyde for 5 min, followed by a 20 min wash with PBS with 0.2% Tween-20 (PBST). The desired primary antibodies for ALX/FPR2 (1:200), p-p65 (1:200), or p-ERK (1:200) were incubated overnight at 4 °C. Then, the cells were washed before being exposed to the FITC-conjugated secondary antibody (1:50) and washed with PBST. The cell nuclei were then stained with DAPI for 5 min. The cells were examined using an inverted digital fluorescent microscope (OLYMPUS, CKX53, Tokyo, Japan).

### 2.7. ELISA

Each experimental group’s culture supernatant was collected. To quantify the levels of pro-inflammatory components, the IL-1β detection kit (Jianglai, Shanghai, China) and IL-6 detection kit (Neobioscience, Shenzhen, China) were used. An Infinite 200Pro microplate reader (Tecan^®^, Grödig, Austria) was used to calculate absorbance.

### 2.8. Statistical Analysis

Data from three independent replicates are presented as the mean ± standard deviation (SD) and were analyzed using GraphPad Prism (version 10.3.1, San Diego, CA, USA). The Shapiro–Wilk and Levene’s tests were used to assess data normality and homogeneity of variances, respectively. For datasets satisfying both assumptions, a two-way ANOVA was performed, followed by Tukey’s post hoc test. Otherwise, the non-parametric Kruskal–Wallis test was used, followed by Dunn’s test with Bonferroni correction. *p* < 0.05 was considered statistically significant.

## 3. Results

### 3.1. Culture and Identification of hPDLCs

**Figure 1:**
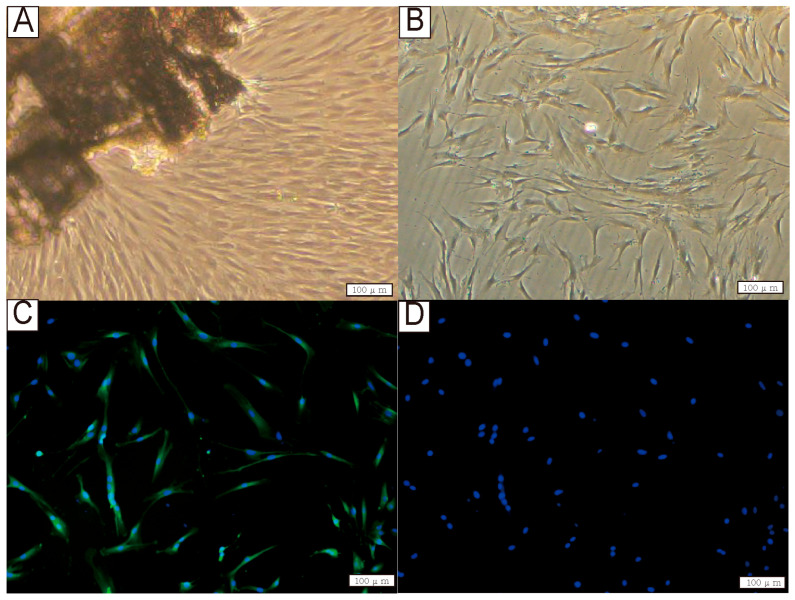
Isolation and phenotypic characterization of primary hPDLCs. (**A**) The hPDLCs grew out from the periodontal ligament tissue. (**B**) The first generation of human periodontal ligament cells. (**C**) Immunocytochemistry staining showed the expression of vimentin (green) in the cytoplasm. (**D**) Immunocytochemistry staining did not show the expression of keratin. Cell nuclei were counterstained with DAPI (blue). All scale bars are 100 μm.

HPDLCs were successfully isolated and then characterized by morphological observation and immunocytochemistry staining. Under light microscopy, hPDLCs displayed typical spindle-shaped fibroblasts (Figure 1A,B). The cells exhibited positive staining for vimentin (Figure 1C) and negative staining for keratin (Figure 1D), which suggested a mesenchymal origin of the primary cultures.

### 3.2. Screening of DEGs

**Figure 2:**
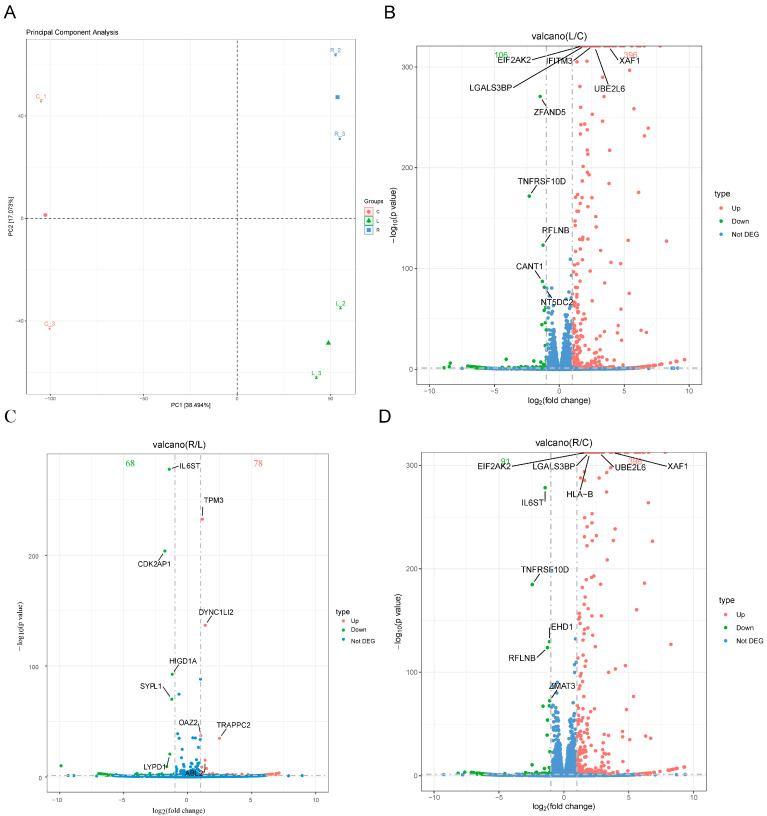
Global clustering and differential expression across conditions. (**A**) PCA of RNA-seq transcriptomic data. Each point represented a sample, and points of the same color were a group. The group centroid is located at the center of the points in the same group. (**B**–**D**) The DEGs were represented by a volcano plot, where each volcano plot was labeled with the top five significantly differentially expressed up-regulated or down-regulated genes. The Red and green dots respectively indicate up-regulated and down-regulated genes, with the adjacent same-colored numbers indicating the total gene count for each category. And the blue dots indicate genes with no significant difference. (**B**) Volcano plot of LPS vs. Control. (**C**) Volcano plot of LPS+RvD1 vs. LPS. (**D**) Volcano plot of LPS+RvD1 vs. Control.

Principal component analysis (PCA) revealed clear segregation among the control (C), LPS (L), and LPS+RvD1 (R) groups, with biological replicates tightly clustered within each group, indicating high data quality and reproducibility (Figure 2A).

Differential expression analysis identified distinct transcriptional signatures across comparisons (Figure 2B–D, Table S2). In the LPS versus control group, 396 genes were upregulated, and 106 genes were downregulated (Figure 2B). Representative changes included upregulated *XAF1* and downregulated *ZFAND5* (Figure 2B). Among the most significantly up-regulated genes, *XAF1* (XIAP Associated Factor 1) participates in cytokine and interferon-stimulated antiviral pathways [31,32]. The DEGs were enriched in interleukin-mediated signaling. Moreover, the significantly down-regulated *ZFAND5* (Zinc Finger AN1-Type Containing 5) has a regulatory function in NF-κB activation and cell death [33,34,35].

For the LPS+RvD1 versus the LPS group, 78 genes were upregulated (Figure 2C). Among them, *IL6ST* (Interleukin 6 Cytokine Family Signal Transducer) was a downregulated gene with significant differences in expression levels. The regulation of immune response, pain control and bone metabolism. In addition, 396 genes were upregulated, and 91 genes were downregulated between the LPS+RvD1 and control (Figure 2D).

### 3.3. GO Analysis

**Figure 3:**
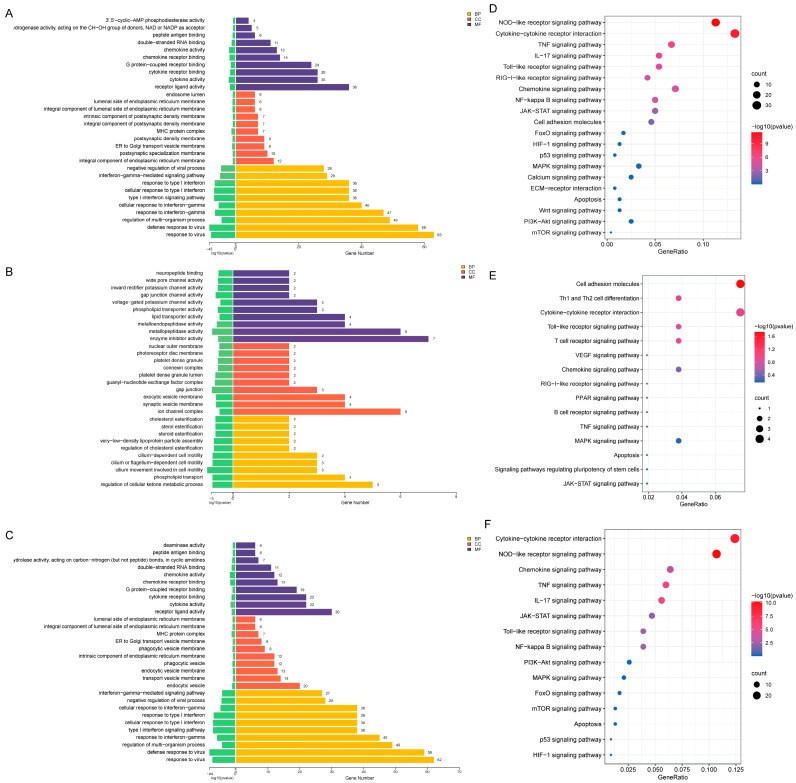
The GO enrichment bar chart of differential genes and bubble map of KEGG pathway enrichment. (**A**) GO analysis of DEGs in LPS vs. Control. The shorter the *p*-value line (green bars) in the figure, the smaller the *p*-value and the more significant the difference. (**B**) GO analysis of DEGs in LPS+RvD1 vs. LPS. (**C**) GO analysis of DEGs in LPS+RvD1 vs. Control. (**D**) Bubble map of the 20 significant pathways in LPS vs. Control. The size of each point corresponds to the number of genes mapped to the pathway, while the color intensity represents the statistical significance level, expressed as −log_10_ (*p*-value) (darker color indicates a smaller *p*-value and higher significance). (**E**) Bubble map of the 15 significant pathways in LPS+RvD1 vs. LPS. (**F**) Bubble map of the 15 significant pathways in LPS+RvD1 vs. Control.

To elucidate the biological functions of these DEGs, GO enrichment analysis was performed (Figure 3A–C), including the three major categories of biological processes (BP), cellular components (CC) and molecular functions (MF) [11]. The top 10 significantly enriched GO terms are presented in bar plots (Figure 3A–C).

Between the LPS and control groups, DEGs were primarily gathered in the following categories (Figure 3A). For the BP category, regulation of multi-organism processes, autoinflammatory and autoimmune diseases were overrepresented [36]. For the CC, regulation of cells, cell parts, organelles, membranes, and major histocompatibility complexes (MHCs) was overrepresented. For the MF, regulation of cytokine activity, cytokine receptor binding and chemokine activity were overrepresented.

Between the LPS+RvD1 and LPS groups, the DEGs were mainly enriched in the following categories (Figure 3B). For the BP, regulation of cellular ketone metabolic process and cell motility were overrepresented. For the CC, regulation of cholesterol esterification, ion channel complex, and synaptic vesicle membrane was overrepresented. For the MF, regulation of enzyme inhibitor activity, lipid transporter activity, and phospholipid transporter activity were overrepresented.

Finally, the enrichment pattern of DEGs between the LPS+RvD1 and control groups was largely similar to that observed between the LPS and control groups (Figure 3C).

### 3.4. KEGG Pathway Analysis

KEGG enrichment revealed that DEGs were overrepresented in pathways related to inflammation, apoptosis, autophagy and oxidative stress.

For LPS versus control (Figure 3D), significantly enriched pathways included the NOD-like receptor signaling pathway, cytokine-cytokine receptor interplay, NF-κB signaling pathway, Toll-like receptor signaling pathway, and MAPK signaling pathway. For LPS+RvD1 versus LPS (Figure 3E), enrichment was observed in cytokine-cytokine receptor interplay, cell adhesion molecules, MAPK signaling pathway, and Toll-like receptor signaling pathway. For LPS+RvD1 versus control (Figure 3F), the most enriched pathways were the NOD-like receptor signaling pathway and cytokine-cytokine receptor interaction.

Most DEGs are mapped to inflammatory mediators, chemokines, and immune-related factors. Considering the results from KEGG analysis (Table S3), subsequent analyses focused on the inflammation-related pathways, including the Toll-like receptor signaling pathway, NF-κB signaling pathway, and MAPK signaling pathway.

### 3.5. Validation of DEGs Data by qRT-PCR

**Figure 4:**
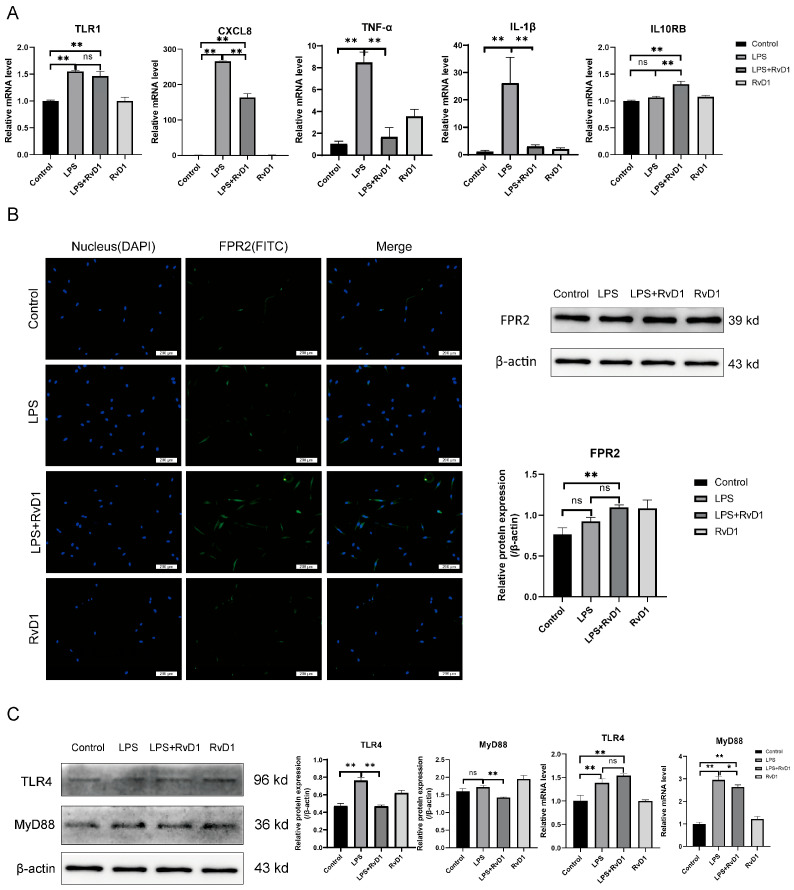
The regulation of RvD1 is mediated by TLR4-MyD88 and activated by ALX/FPR2 receptors. (**A**) The analysis of the mRNA was undertaken using qRT-PCR, including *TLR1*, *CXCL8*, *TNF-α*, *IL-1β*, and *IL10RB*. All values are the mean  ±  SD. (**B**) The effects of RvD1 on hPDLCs are ALX/FPR2 receptor dependent. Immunofluorescence images showing ALX/FPR2 expression (green) in hPDLCs, with nuclei counterstained by DAPI (blue). Scale bar is 200 μm. Western blotting and quantification results for ALX/FPR2 protein levels. (**C**) The effect of RvD1 on the expression of TLR4 and MyD88. WB of TLR4 and MyD88 protein expression level. qRT-PCR of TLR4 and MyD88 mRNA expression level. (** *p* < 0.01; * *p* < 0.05; ns, not significant).

To validate the RNA-seq results, several representative DEGs were selected for qRT-PCR analysis. In the LPS versus control comparison, the levels of *TLR1*, *CXCL8*, *TNF-α*, and *IL-1β* were significantly upregulated. In contrast, in the LPS+RvD1 versus LPS comparison, the pro-inflammatory genes *CXCL8*, *TNF-α*, and *IL-1β* were significantly downregulated, while the anti-inflammatory gene *IL10RB* was markedly upregulated (Figure 4A). The findings demonstrated a high degree of consistency with RNA-seq data, further reinforcing the reliability and robustness of the transcriptomic analysis.

### 3.6. Impact of RvD1 on hPDLCs Depends on ALX/FPR2

To investigate whether the impact of RvD1 on hPDLCs is mediated through ALX/FPR2, IF and WB analysis were used to observe the expression of ALX/FPR2 (Figure 4B). The results of WB showed that ALX/FPR2 expression in the LPS group exhibited a marginal increase compared to the control group, but the difference was not statistically significant. In the LPS+RvD1 versus LPS comparison, RvD1 treatment increased the level of the ALX/FPR2 receptor, but the difference was not statistically significant. In the results of IF, the strongest ALX/FPR2 signal was in the LPS+RvD1 group, followed by the LPS group, while the control group showed the lowest signal. These IF results aligned with the trend observed in WB analysis.

### 3.7. Impact of RvD1 on Expression of TLR4 and MyD88

In comparison with the control group, LPS increased TLR4 expression. RvD1 decreased the expression level of TLR4 in proteins, but RvD1 did not decrease the mRNA expression level of *TLR4*. The qRT-PCR and WB results indicated that RvD1 significantly down-regulated the level of MyD88 in hPDLCs (Figure 4C).

### 3.8. Verify the Function of NF-κB Signaling Pathway Under RvD1 by IF

**Figure 5:**
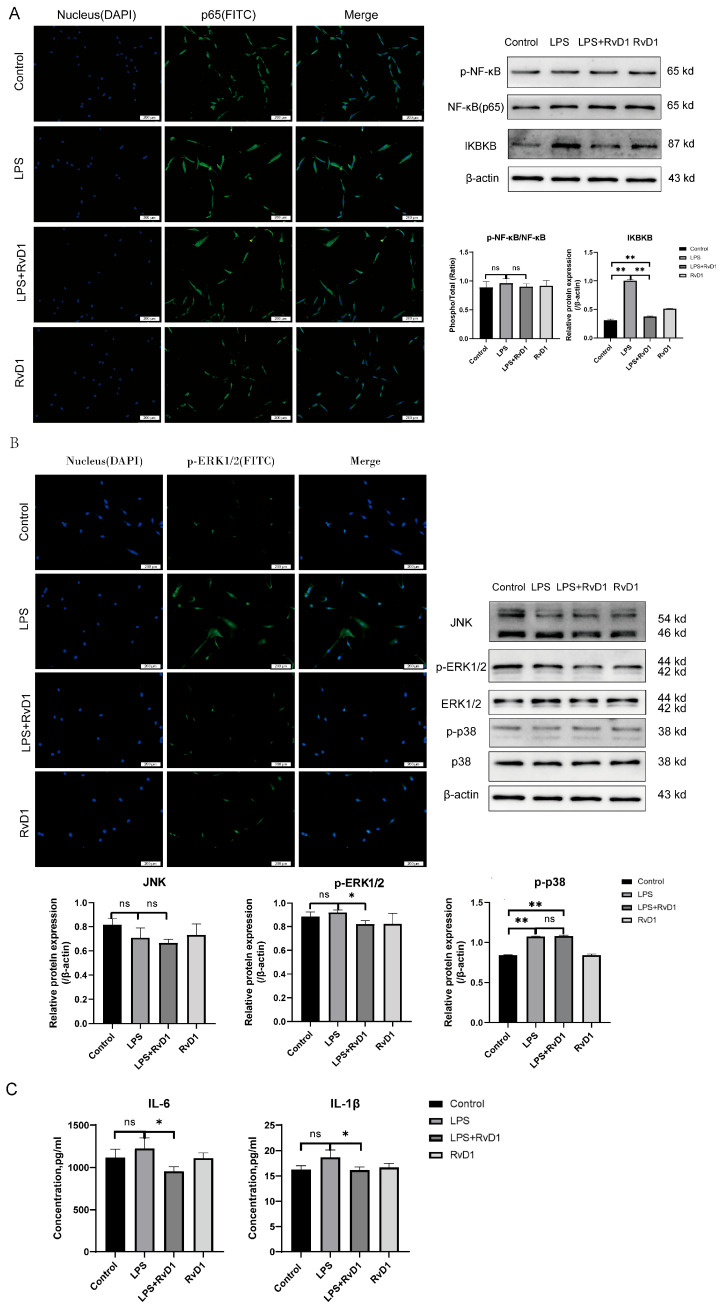
RvD1 exerts its impact by regulating the NF-κB and MAPK signaling pathways. (**A**) RvD1 regulates the nuclear translocation of NF-κB. Compared to stimulation with LPS alone, the addition of RvD1 reduced the nuclear accumulation of NF-κB p65 (green). Nuclei are shown in blue (DAPI). Scale bar is 200 μm. WB of the NF-kB and IKBKB were analyzed. (**B**) IF of p-ERK. RvD1 inhibited the LPS-induced expression of p-ERK (green). The cell nucleus was counterstained with DAPI (blue). Scale bar is 200 μm. The banding pictures of JNK, p-ERK, ERK, p-p38, p38 and β-actin. WB analysis of JNK, p-ERK and p-p38. (**C**) Detection of IL-6 and IL-1β via ELISA (** *p* < 0.01; * *p* < 0.05; ns, not significant).

To explore whether the impact of RvD1 was mediated through regulation of the NF-κB pathway, the NF-κB and IKBKB protein levels were determined by WB. The IKBKB protein level significantly increased with LPS stimulation but decreased with RvD1 treatment (Figure 5A). The protein level changes in phosphorylated p65 were consistent with the results of IKBKB, but there was no statistically significant difference. IF results showed that NF-κB was activated and translocated to the cell nucleus under LPS stimulation. Notably, RvD1 decreased the expression of nuclear NF-κB(p65).

### 3.9. RvD1 Exerts Its Impact by Regulating the MAPK Signaling Pathway

To assess MAPK activation downstream of TLR4–MyD88, the phosphorylation states of ERK and p38, and the level of JNK, were evaluated by WB and IF. LPS exposure produced an upward trend in p-ERK that did not reach statistical significance, whereas RvD1 treatment attenuated p-ERK (Figure 5B).

Total JNK levels showed no significant change across groups. By contrast, p-p38 was significantly increased after LPS stimulation, and RvD1 did not reduce p-p38 relative to the LPS group (Figure 5B).

### 3.10. Detection of Inflammatory Cytokine Levels

In comparison with the control group, IL-6 and IL-1β expression showed a tendency to increase in the LPS group but did not show statistical differences. However, in comparison with the LPS group, the levels of IL-6 and IL-1β were considerably diminished in the LPS+RvD1 group (Figure 5C).

## 4. Discussion

This study tested the hypothesis that RvD1 regulates the inflammatory and infection-stimulated responses of hPDLCs to restore the homeostasis of periodontal tissues. Integrated transcriptomic profiling and orthogonal validation indicate that RvD1 acts primarily through the TLR4–MyD88–NF-κB axis and selectively modulates MAPK signaling (notably ERK), thereby attenuating pro-inflammatory signaling within the periodontal microenvironment.

Several studies have been conducted on neutrophils and monocytes to research the effect of ALX/FPR2 as a key receptor in mediating RvD1 [37,38]. This receptor promotes anti-inflammatory actions and pro-resolution functions after recognizing RvD1 [39,40]. Our findings demonstrated a trend in the increased expression of the ALX/FPR2 receptor following LPS stimulation. These findings were in line with the tendency shown by human polymorphonuclear leukocytes, which express FPR2/ALX more when exposed to pro-inflammatory stimuli [41]. In this work, the effects of RvD1 were explored in vitro, and there was an accompanying rise in FPR2/ALX expression levels. This demonstrated that RvD1 interacted with the ALX/FPR2 receptor to trigger the process of resolving inflammation. Taken together, RvD1 exerts its pro-resolving effects at FPR2/ALX receptors via interacting with ALX and GPR32 [39,42].

**Figure 6:**
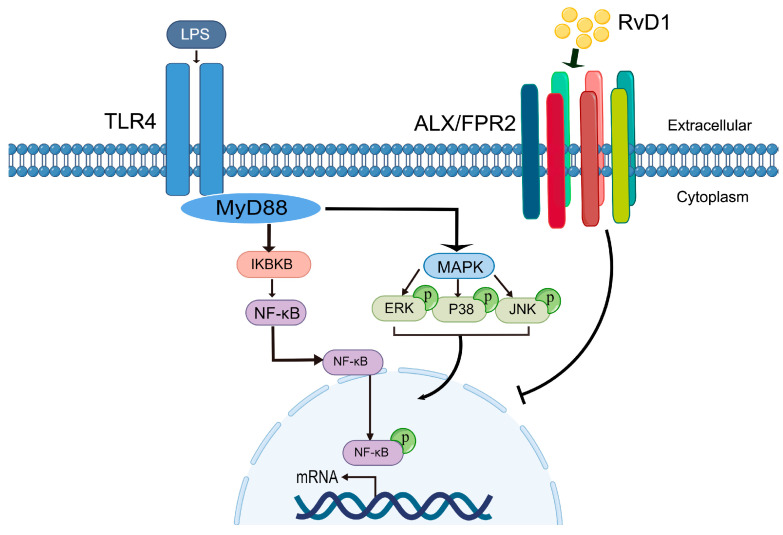
Schematic of RvD1 regulating the inflammatory response of hPDLCs stimulated by LPS. Stimulation of TLR4 by LPS activates the downstream TLR4-MyD88-MAPK signaling pathway and the TLR4-MyD88-NF-κB signaling pathway. RvD1 modulates inflammation by regulating TLR-like receptor-mediated signaling and binding to ALX/FPR2 receptors on cell membranes.

TLR4 is activated by LPS, triggering nonspecific immunological reactions. MyD88-dependent or MyD88-independent signaling pathways are present downstream of TLR4 [43]. Among these, activation of the MyD88-dependent pathway causes NF-κB and MAPK signaling pathways to be activated (Figure 6). Adaptor protein MyD88 initiates the MyD88-mediated pathway [43,44]. The IL-1 receptor-associated kinase (IRAK) 4 is then enlisted by MyD88, and IRAK1 and IRAK2 are subsequently activated [45]. These kinases must be activated in order to activate MAPK and NF-κB [46,47]. IRAKs then detach from the MyD88-IRAK complex and engage with TNF receptor-associated factor 6 (TRAF6), leading to the recruitment and activation of transformation growth factor-β-activated kinase 1 (TAK1, encoded by the *MAP3K7* gene) [48]. Once activated, TAK1 in turn phosphorylates and activates downstream components of the NF-κB and MAPK signaling pathways [47]. In our study, TLR4 and MyD88 expressions increased under LPS stimulation, as demonstrated by WB and qRT-PCR. However, RvD1 reduced TLR4 and MyD88 protein expression and decreased *MyD88* mRNA expression. These results indicate that RvD1 attenuates the response to inflammation by modulating the expression of TLR4 and MyD88. Moreover, our results also showed that *TLR4* mRNA expression was unaffected by the action of RvD1. This could be because of the micro-RNAs’ control on toll-like receptor signaling. This is consistent with the established role of microRNAs (miRNAs), which primarily reduce target mRNA levels by binding to the 3′ untranslated region (3′ UTR), leading to mRNA degradation [49,50]. Previous studies indicate that RvD1 can regulate specific miRNAs that function as negative regulators of inflammatory signaling [51]. Furthermore, previous studies have demonstrated that RvD1 reduces TLR4 protein expression without affecting its mRNA levels. In a myocardial infarction model, RvD1 pretreatment significantly decreased TLR4 protein expression [52]. Similarly, in lung ischemia–reperfusion injury, RvD1 treatment reduced TLR4 protein levels without altering its mRNA expression [53].

The transcription factor NF-κB regulates immunity and inflammation. Excitation of NF-κB occurs by IKK-mediated phosphorylation of the inhibitory protein IκB. IKKα, IKKβ, and IKKγ are the three subunits that make up the IKK complex [54,55]. Among them, IKKβ is the primary target for stimuli that cause inflammation. During the resting state, NF-κB is normally confined to the cytoplasm due to its binding to the inhibitor protein IκB. NF-κB dimers are released and moved to the nucleus when IKK phosphorylates inhibitor protein IκB, leading to its degradation [56,57]. Our findings showed that LPS stimulates hPDLCs, the activated IKKβ eventually increases, and then the NF-κB is activated, which then translocates into the nucleus. However, RvD1 suppressed the expression of IKKβ and the nuclear translocation of NF-κB. These findings suggest that RvD1 suppressed the activated NF-κB signaling pathway to a certain extent.

The MAPK signaling pathway is involved in the production of pro-fibrotic and pro-inflammatory mediators, including JNK, ERK, and p38 [58]. The TLR4-mediated signaling pathway, which is MyD88-dependent, activates TAK1. After that, active TAK1 activates MAPKs [12,59]. The signaling from the activated MAPKs induces the transcription factor AP-1, which aids in the production of pro-inflammatory cytokines [60]. Our findings indicated an upward trend in the levels of p-ERK protein expression, but no significant statistical change was seen. RvD1 could inhibit the activation of p-ERK. RvD1 and other SPMs have been reported to activate phospholipases and activate downstream signaling molecules ERK1/2 and calcium ions [61,62,63]. Consistent with earlier studies, the present results demonstrate that RvD1 suppresses LPS-induced ERK phosphorylation. In human airway epithelial cells, RvD1 inhibited mimic poly (I: C) induced ERK activation through interference with TAK1 signaling, thereby attenuating downstream inflammatory responses [53]. Likewise, RvD1 reduced Ang II-induced hypertension and vascular remodeling in vascular smooth muscle cells through suppression of the RhoA/MAPK pathway, including reduction in ERK phosphorylation [64]. The concordance of these independent observations with our results supports the notion that ERK inhibition may represent a conserved mechanism through which RvD1 exerts its anti-inflammatory actions across diverse cell types.

In contrast, neither LPS stimulation nor RvD1 regulation produces a significant change in total JNK protein levels, which differs from several other cellular models. For example, RvD1 was reported to inhibit hemoglobin-induced JNK phosphorylation in microglial cells and suppress IL-1β–induced JNK1/2 activation in osteoarthritis chondrocytes [21,65]. In agreement with the present results, however, one study using LPS-stimulated human monocytes also found that resolvins, including RvD1, did not influence SAPK/JNK phosphorylation [51]. Together, these findings highlight the cell-type-dependent variation in JNK pathway responses to RvD1 and underscore the importance of cellular context in interpreting signaling modulation.

However, following LPS stimulation, the expression of p-p38 was noticeably elevated, and RvD1 was unable to lower this expression. It showed that while 12 h of RvD1 impact might ineffectively lower the phosphorylation, 24 h of LPS stimulation could promote TLR4-mediated p38 phosphorylation. Previous research indicated that RvD1 regulates inflammatory responses in hPDLCs by activating p38 protein. The findings from our experimental model differ from those reported in several previous studies, such as microglial cells, osteoarthritis chondrocytes [21,25,65]. However, growing evidence indicates that p38 signaling can play distinct roles based on the cellular and inflammatory microenvironment. For instance, in human visceral adipose tissue, RvD1 enhanced p38 phosphorylation and promoted the expression of anti-inflammatory mediators such as heme oxygenase-1 [66,67]. The dual nature of p38 signaling, which enables its participation in both pro-inflammatory and pro-resolving processes, may explain why RvD1 did not suppress p38 activation in our experimental system. LPS-induced expressions of IL-6 and IL-1β exhibited an upward trend without reaching statistical significance. The LPS concentration employed in this study (0.1 μg/mL) was relatively low compared to concentrations used in other studies (ranging from 0.5 to 2.0 μg/mL) that demonstrated significant cytokine elevation [68,69,70]. This suggests that the LPS stimulation in our experimental setting may have triggered a milder yet biologically relevant inflammatory response. Notably, RvD1 significantly suppressed both cytokines, an effect consistent with other anti-inflammatory agents, underscoring its therapeutic potential in early or mild inflammation [71].

This study has primarily explored the underlying mechanisms through in vitro experiments. To further validate the results, we plan to conduct organ-on-a-chip models and animal experiments in follow-up work and report those findings in subsequent publications.

These findings suggested that the TLR4-MyD88-MAPK and TLR4-MyD88-NF-κB signaling pathways were most likely regulated by RvD1 to control the LPS-induced inflammatory response in hPDLCs.

## 5. Conclusions

In this work, RNA-seq technology was employed to conduct a comprehensive gene expression pattern analysis of the role of RvD1 in regulating hPDLCs’ inflammatory response. It has been confirmed that RvD1 regulates the inflammatory response of LPS-stimulated PDLCs by modulating the TLR4-MyD88-MAPK and TLR4-MyD88-NF-κB signaling pathways. Therefore, this study demonstrated the potential role of RvD1 in the treatment of periodontitis.

## Data Availability

The datasets used during the work are available from the corresponding author. The raw RNA-sequence data for this article were deposited in the National Center for Biotechnology Information, National Library of Medicine. The BioProject number was PRJNA1307989. The link is https://dataview.ncbi.nlm.nih.gov/object/PRJNA1307989?reviewer=ep6l0614buoj2j8qn701ckl318 (accessed on 30 November 2025).

## Supplementary Materials

The following supporting information can be downloaded at https://www.mdpi.com/article/10.3390/biomedicines13123038/s1. Table S1: Primer sequences used for real-time qRT-PCR. F, Forward; R, Reverse. Table S2: Summary of DEGs in each group. Table S3: KEGG pathway enrichment of DEGs.

## Abbreviations

The following abbreviations are used in this manuscript:

RvD1	Resolvin D1
SPMs	Specialized proresolving lipid mediators
hPDLCs	Human periodontal ligament cells
qRT-PCR	Quantitative real-time PCR
GO	Gene Ontology
KEGG	The Kyoto Encyclopedia of Genes and Genomes
DEGs	Differentially expressed genes
TLR4	Toll-like receptor 4
MyD88	Myeloid differentiation factor 88
NF-κB	Nuclear factor-κB
MAPK	Mitogen-activated protein kinase
IF	Immunofluorescence
IL	Interleukin
ELISA	Enzyme-linked immunosorbent assay
ERK	Extracellular signal-regulated kinase
JNK	C-Jun NH2-terminal kinase
LPS	Lipopolysaccharide
TLRs	Toll-like receptors
RNA-seq	RNA sequencing
PCA	Principal component analysis
BP	Biological processes
CCs	Cellular components
MFs	Molecular functions
IRAK4	IL-1 receptor-associated kinase 4
TRAF6	TNF receptor-associated factor 6
TAK1	Transforming growth factor-β-activated kinase 1
